# PHYSICAL FITNESS, FATIGABILITY, AND LIFE-SPACE MOBILITY: DIFFERENCES BY NEIGHBORHOOD CONTEXT

**DOI:** 10.1093/geroni/igae098.0237

**Published:** 2024-12-31

**Authors:** Kyle Moored, Kate Duchowny, Andrea Rosso, Peggy Cawthon, Michelle Carlson, Nancy Glynn

**Affiliations:** Johns Hopkins Bloomberg School of Public Health, Baltimore, Maryland, United States; University of Michigan, Ann Arbor, Michigan, United States; University of Pittsburgh, Pittsburgh, Pennsylvania, United States; California Pacific Medical Center, San Francisco, California, United States; Johns Hopkins University, Baltimore, Maryland, United States; University of Pittsburgh School of Public Health, Pittsburgh, Pennsylvania, United States

## Abstract

Physical capacity (e.g., objective fitness, perceived fatigability) contributes independently to life-space mobility, the extent and independence of travel within the community. This relationship may differ by neighborhood context, where higher physical capacity may be especially necessary to overcome environmental barriers to mobility. We examined baseline associations between physical capacity and life-space mobility in community-dwelling older adults, and whether these associations were moderated by perceived neighborhood factors. Participants were from the Study of Muscle, Mobility and Aging (SOMMA; N=775, Mean±Standard Deviation age=76.1±4.9). Life-space mobility was self-reported using the Life-Space Assessment (higher=greater life-space, 83.6±19.3). Physical capacity measures included maximal treadmill cardiorespiratory fitness (VO2peak, 20.3±4.8 mL/min/kg) and perceived physical fatigability (Pittsburgh Fatigability Scale (PFS) Physical score, higher=more fatigability, 15.4±8.5). Self-reported neighborhood physical disorder (e.g., trash/vandalism) and social cohesion (e.g., neighborhood trust) scores were stratified into tertiles. Linear regressions estimated associations adjusted for demographic, health, and socioeconomic confounders. Each 1-SD higher VO2peak was associated with 2.5-point higher (95% CI: 0.84,4.2) life-space score, with associations significantly greater for the highest physical disorder tertile (b=3.8, 95% CI: 1.5,6.2, p-interaction=.018) and lowest social cohesion tertile (b=5.7, 95% CI: 2.9,8.6, p-interaction=.046). Each 1-SD higher PFS Physical score was associated with 3.2-point lower (95% CI: -4.6,-1.7) life-space score, with no differences by neighborhood measures (p-interactions>.15). Unlike perceived physical fatigability, the impact of objective fitness on life-space mobility may be greatest for those living in more disordered and less socially integrated neighborhoods. Findings reinforce the importance of considering individual-level contributors to life-space mobility within the environmental context (i.e., person-environment fit).

